# Wealth related inequality in women and children malnutrition in the state of Chhattisgarh and Tamil Nadu

**DOI:** 10.1186/s40795-022-00580-1

**Published:** 2022-08-22

**Authors:** P. Shirisha, V. R. Muraleedharan, Girija Vaidyanathan

**Affiliations:** Humanities and Social Sciences Block, Indian Institute of Technology, Madras, India

**Keywords:** Malnutrition, Stunting, Wasting, Underweight, NFHS, Anaemia

## Abstract

**Background:**

Child and maternal malnutrition are the most serious health risks in India, accounting for 15% of the country’s total disease burden. Malnutrition in children can manifest as ‘stunting’ (low height in relation to age) or ‘wasting’ (low weight in relation to height) or both and underweight or obesity among women. Other nutritional indicators show that India lags behind, with high levels of anaemia in women of reproductive age. The study aims to analyse the wealth related inequalities in the nutrition status among women and children of different wealth quintiles in a high focus state (Chhattisgarh; CG) and a non-high focus state (Tamil Nadu; TN) in India.

**Methods:**

We used National Family Health Survey-3rd (2005–06) & 4th (2015–16) to study the trends and differentials of inequalities in the nutrition status. We have used two summary indices. - absolute inequalities using the slope index of inequality (SII), and relative inequalities using the concentration index (CIX).

**Results:**

There is reduction in wealth related inequality in nutrition status of women and children from all wealth quintiles between 2005–06 and 2015–16. However the reduction in inequality in some cases such as that of severe stunting among children was accompanied by increase among children from better off households The values of SII and CIX imply that malnutrition except obesity is still concentrated among the poor. The prevalence of anaemia (mild, moderate and severe) has reduced among women and children in the past decade. The converging pattern observed with respect to prevalence of mild and moderate anaemia is not only due to reduction in prevalence of anaemia among women from poor households but an increase in prevalence in rich households.

**Conclusion:**

Malnutrition remains a major challenge in India, despite encouraging progress in maternal and nutrition outcomes over the last decade. Our study findings indicate the importance of looking at the change in inequalities of nutrition status of women and children of different wealth quintiles sub nationally. Given the country’s rapidly changing malnutrition profile, with progress across several indicators of under nutrition but rapidly rising rates of overweight/obesity, particularly among adults, appropriate strategies needs to be devised to tackle the double burden of malnutrition.

## Introduction

Maternal and child malnutrition are the most serious health risks in India, accounting for 15% of the country’s total disease burden [1]. The recently released study on disease burden identified malnutrition as one of three national emergencies [2]. It was the leading cause of death among under 5 children in every Indian state, accounting for 68.2% of all under-five deaths and the leading cause of health loss for all ages, accounting for 17.3% of total disability-adjusted life years. The short term consequences of undernutrition are morbidity, mortality, and disability [3]. While, the chronic forms of malnutrition (stunting) during early childhood affects brain structure and function with long-term negative consequences such as reduced mental ability and learning capacity, poor school performance, lower earnings [4]. Other nutritional indicators in which India lags behind are undernourishment and high levels of anaemia in women of reproductive age. A quarter of Indian women of reproductive age are malnourished, with a BMI of less than 18.5 kg/m2 [5]. It is well understood that an undernourished mother will inevitably give birth to an undernourished child, perpetuating an intergenerational cycle of malnutrition.

### Intergenerational cycle of malnutrition: a vicious cycle leading to child growth failure

The intergenerational cycle is the path through which a low-birth-weight baby becomes a stunted child, a stunted adolescent and a malnourished woman who in turn, has another low-birth-weight baby [6, 7]. The vicious cycles of malnutrition operates by affecting in utero or infants, when proper care of food and health isn’t taken care during that period, as a result the child’s development is not to his/her potential, physically as well as mentally. The most disadvantaged section includes girls, who then have poor weight gain during pregnancy owing to various social and environmental factors such as poverty resulting in inappropriate food and health care. Therefore children and women’s nutrition status are linked. A recent study from India using all four rounds of National Family Health Survey examined that the children whose mothers are underweight (with a body mass index less than 18.5 kg/m^2^) are more likely to be stunted, wasted and underweight than those children whose mothers have normal BMI or are obese [8]. On the other hand overweight or obesity among women is associated with poor pregnancy outcomes. Obese mothers are more likely to develop gestational diabetes and pre-eclampsia, potentially exposing the fetus to a harmful intrauterine environment [9]. Finding from observational studies strongly implies that maternal obesity before pregnancy and excessive gestational weight gain are associated with an increased risk of foetal pregnancy complications and adverse childhood cardiometabolic, respiratory, and cognitive-related health outcomes [10]. Maternal obesity is associated with fetal macrosomia, which leads to increased neonatal and maternal morbidity [9]. Another study found that there is a 264% increase in the odds of child obesity when mothers are obese prior to conception [11]. Similar to undernutrition and overweight or obesity, the risk of anaemia among children is associated with the mother’s nutritional status. There is a greater prevalence of severe, moderate and mild anaemia among children belonging to moderately anaemic mothers [8, 12]. Anaemia has been linked to poor overall cognitive development and physical growth in children, as well as morbidity from infectious diseases [13–15]. Although there has been a progressive reduction in anaemia from 1992 to 93 to 2015–16 (National Family Health Survey (NFHS)), the decline has been too slow. The latest data (NFHS-5;2019–21) reports almost 10% increase in prevalence of child anaemia in India (67%) and worsening of anaemia status (53 to 57%). Hence, tracking the progress of women‘s nutrition is pivotal if the burden of child malnutrition is to be reduced.

Undernourishment affects all segments of the Indian population [16]. A number of studies across and within the countries report strong and significant association of malnutrition with economic status. Several of which reported that a significant correlation exists between the socioeconomic gradient and maternal and child nutrition status. With malnutrition being primarily concentrated among low socioeconomic status households [3, 17–26]. Therefore the rationale of our study is to analyse the change in inequality with regards to women and child malnutrition at subnational levels. India is a populous country with 29 states and seven union territories which are at various levels of development, thus with uneven distribution of health risks and their consequences [27–29]. Therefore, variations also exist with respect to the child growth failure (CGF) indicators (stunting, wasting and underweight) and women’s nutrition indicators between states [30]. The southern states- Kerala, Tamil Nadu are recognized as “positive deviants” in some nutrition-related studies compared to northern states [31]. Few studies have assessed the extent of inequality in nutrition status among women and children at national as well as district level [30, 32]. However, only one study analysed the changes in socio-economic inequality of children as well as adult undernutrition in relation to the areas of residence (urban/rural) [33]. To the best of our knowledge there has been no study which has analysed and compared the trends in inequalities of women and child malnutrition and anaemia (severe and overall) in a high focus (Chhattisgarh) and non-high focus (Tamil Nadu) state of India.^1^ In this study we are analysing the trends at all India level, and comparing it with trends in a high focus and a non-high focus state. We have selected CG as it is a representative of high-focus states, while Tamil Nadu (TN) which is at 2nd spot among the non-high focus states, if not at the top (with Kerala being an outlier with respect to the health outcomes). Another reasons being the authors’ familiarity with the respective states, PS is familiar with Chhattisgarh while VRM and GV are quite familiar with the state of Tamil Nadu. Hence in this study we aim to assess the trends of wealth related inequalities existing with respect to nutrition indicators (BMI for women; stunting, wasting and underweight for children) and different forms of anaemia (mild, moderate and severe) among women and children in Tamil Nadu (TN) and Chhattisgarh (CG). By conducting a trend analysis of the magnitude of inequality in these states, we can understand if the gap between the poor and the rich with regards to the nutrition status and anaemia has increased (worsened) or reduced (improved) between 2005 and 06 and 2015–16. A study of this nature is of importance as health policy at subregional level could be designed for effective interventions. Therefore, assessing the inequality in the prevalence of malnutrition by state development may highlight different immediate health policy priorities between less and more developed states.

## Method of analysis

### Data sources

The two rounds of nationally representative survey: National Family Health Survey (NFHS-3 and NFHS-4, conducted in 2005–06 and 2015–16 respectively) form the data source for this analysis. NFHS provides state and national level information on fertility, family planning, infant and child morbidity and mortality, maternal and reproductive health, nutritional status of women and children, and the quality of the health services in all 29 states as well as in the union territories. It is the Indian equivalent of Demographic Health Survey (DHS). In NFHS-4 (2014–16), the data was collected for 6,01,509 households while in the 3rd (2005–06) 1,09,041 households were covered. NFHS uses multistage sampling techniques, so it is important to use appropriate weights that make the estimates to be representative and comparable in both rounds. The details of the sampling weights are given in the NFHS reports (IIPS, 2006; International Institute for Population Sciences (IIPS) and ICF, 2017) [5, 35].

### Ethics statement

In the original household survey, a written informed consent was obtained from the participants for participation in this survey before commencement of the interview and best efforts were made to maintain privacy. The data are available in the public domain without any personal identifier.

### Indicators of undernutrition

For children, standard indices of physical growth related to nutritional status are height-for-age (stunting), weight-for-height (wasting), and weight-for-age (underweight) z-scores. A child who is below minus two standard deviations (− 2 SD) from the median of the WHO reference population in terms of height-for-age is considered short for his/her age or stunted and severely stunted (below − 3 SD). Stunting reflects the cumulative effect of chronic malnutrition. While the children whose weight-for-height is below − 2 SD from the median of the WHO reference population are considered too thin for his/her height or wasted while those below − 3 SD are considered as severely wasted. Wasting is a condition reflecting acute or recent nutritional deficit or a recent illness. Weight-for-age is a composite index of stunting and wasting and is a good indicator to monitor nutritional status over time [36]. The height and weight measures of women were used to calculate body mass index (BMI) in kg/m^2^. Underweight was defined as BMI < 18.5 kg/m^2^ and overweight as BMI ≥25 kg/m^2^.This categorisation is based on the WHO’s recommended cut-offs for BMI classification [37]. We have also assessed the disparities in prevalence of anaemia (mild, moderate and severe) among children and women belonging to different wealth quintiles. To classify different forms of anaemia among women and children, we have used the cut off values of haemoglobin provided in Table 1.

**Table 1:** Classification of anaemia among women (15–49 years) and children (under-5)

Classification	Normal	Mild	moderate	Severe
Children	12 g/dl	10–11.9 g/dl	7–9.9 g/dl	< 7 g/dl
Women (15–49 years)	11 g/dl	10–10.9 g/dl	7–9.9 g/dl	< 7 g/dl

Source: NFHS-3 (2005–06) and NFHS-4 (2015–16) reports

We have recalculated indicators from the original survey data retrieved from the DHS website and estimated different indicators to adjust for the survey design, including weight and cluster sampling (IIPS, 2006; International Institute for Population Sciences (IIPS) and ICF, 2017). The results obtained were compared with the official report of CG, TN state and All India report of both rounds (NFHS-3 & 4) and were found to be consistent.

There is near consensus in the literature that research studies should present both relative and absolute measures to capture the magnitude of inequality [38, 39]. The existing methods of absolute and relative measures of inequality have certain limitations [40] and to overcome these limitations, Harper and Lynch came up with a set of sophisticated measure of inequality: the slope index of inequality (SII) as an indicator of absolute inequality and concentration index (CIX) or the relative index of inequality (RII) as an indicator of relative inequality. The SII is less affected by the sample size in each socioeconomic group. For measuring the SII, linear regression of dependent variable (intervention) over the wealth index (explanatory variable) gives a slope, which provides an absolute measure of the difference (in the intervention coverage) between the highest (score of 1) and the lowest (score of 0) values of the socioeconomic indicator rank. CIX is related to the Gini coefficient which is a well-known measure of wealth/income concentration. Individuals are ranked according to their socioeconomic status on the x-axis while cumulative intervention coverage is plotted on the y-axis. The distance between the curve and the diagonal gives the concentration of wealth. It shows to what extent is an intervention concentrated among the wealthiest or poorest. The CIX and SII values (when multiplied by 100) varies from (−)100 to 100 percentage points. A positive value implies a pro-rich pattern, that is, prevalence of malnutrition is higher among the women, children from rich households. A negative value implies pro-poor pattern i.e. prevalence of malnutrition is higher among women and children belonging to poor households.

To assess the magnitude of inequality in the nutrition status of women and children across households from different economic strata, we have used the data on wealth quintile provided by NFHS. In NFHS for estimation of the wealth quintile, the households were given scores based on the assets they possessed and their housing characteristics. The score of each household asset is derived using principal component analysis (PCA) [41]. The households are divided into five equal categories, each having 20 percentage of population or an equal number of individuals [5]**.** So, as a result, there are five quintiles which are: poorest (Q1), poorer (Q2), middle (Q3), richer (Q4), richest (Q5). Following table (Table 2) shows the change in wealth gap between the households belonging to different wealth quintiles across both the rounds of NFHS at all India level.

**Table 2:** Percentile-wise cut off values of household wealth index score for NFHS-3 (2005–06) and NFHS-4(2015–16)

Percentile of households	Cut off values for wealth index score (NFHS-3)	Cut off values for wealth index score (NFHS-4)
Below 20th percentile (poorest)	−1.77 to − 1.01	−2.4 to − 0.93
20th to 40th percentile (poorer)	Above − 1.01 to − 0.41	Above − 0.93 to − 0.266
Above 40th to 60th percentile (middle)	Above − 0.41 to 0.26	Above − 0.266 to 0.3948
Above 60th and 80th percentile (richer)	Above 0.26 to 1.02	Above 0.39 to 1.07
Above 80th percentiles (richest)	Above 1.02 to 2.48	Above 1.07 to 3.00
Median	−0.084	0.063

Source: NFHS-3 & 4 reports accessed from https://dhsprogram.com/programming/wealth%20index/India%20DHS%202005-06/india%202005-06.pdf and https://dhsprogram.com/programming/wealth%20index/India%20DHS%202015-16/IndiaNFHS4.pdf respectively

The minimum cut off values for the poorest households has become more negative from NFHS-3(2005–06) to NFHS-4 (2015–16), however the maximum cut off values for the richest households has increased. Therefore, the wealth gap between the poorest and richest households has increased.

## Results

**Fig. 1:**
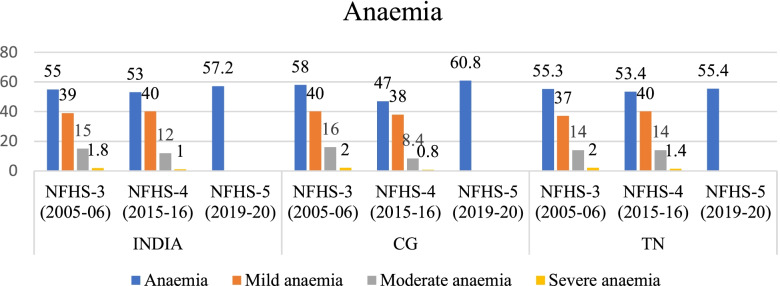
Trends in prevalence of anaemia among women (15–49 years) in India, CG, TN from NFHS-3 (2005–06) to NFHS-5 (2019–20). Source: Data from NFHS-3, NFHS-4 and NFHS-5 fact sheet of India, CG and TN

Using the SII and CIX values we have assessed the trends of inequality in nutrition status among women and children of different wealth quintiles in India, CG and TN (Tables 3 & 4). In this section we use equiplots (created with the help of *equiplots creator tool* developed by International Centre for Equity in Health, Pelotas) to present our results and show the disparities in the nutrition status among women and children belonging to different wealth quintiles -poorest (Q1), poorer (Q2), middle (Q3), richer (Q4), richest (Q5) [42]. The trend shows that inequalities in prevalence of undernutrition and different forms of anaemia (mild, moderate and severe) has reduced across women of different wealth quintiles however it is still concentrated among the children and women from poor households. But, anaemia continues to be a ‘severe public health’ among women in CG, TN and India according to WHO cut off values of public health significance for anaemia (Fig. 1 & Table 3).

**Table 3:** Cut-off values for public health significance (for anaemia)

Indicator	Prevalence cut-off values for public health significance	
Anaemia	< 5%:	No public health problem
	5–19%:	Mild public health problem
	20–39%:	Moderate public health problem
	≥ 40%:	Severe public health problem

Source: WHO. Global health observatory (GHO) data repository, accessed from https://www.who.int/data/nutrition/nlis/info/anaemia

**Table 4:** Trends in wealth related absolute inequality (SII) in prevalence of malnutrition and anaemia among women

	India	India	Change in absolute inequality**	CG	CG	Change in absolute inequality**	TN	TN	Change in absolute inequality**
	NFHS-3 (2005–06)*	NFHS-4 (2015–16)*		NFHS-3 (2005–06)*	NFHS-4 (2015–16)*		NFHS-3 (2005–06)*	NFHS-4 (2015–16)*	
Thin	−0.39	− 0.28	− 0.09	−0.40	− 0.25	−0.15	− 0.41	− 0.19	− 0.22
Normal	0.02	− 0.08	− 0.10	0.14	− 0.06	− 0.20	− 0.09	− 0.12	0.03
Overweight/obesity	0.40	0.36	−0.04	0.34	0.36	−0.02	0.50	0.30	−0.2
Anaemia	− 0.21	− 0.13	− 0.08	− 0.27	− 0.23	− 0.04	− 0.15	− 0.09	− 0.06
Mild Anaemia	−0.12	− 0.05	− 0.07	− 0.14	−0.15	0.01	−0.05	− 0.03	− 0.02
Moderate Anaemia	− 0.08	− 0.04	− 0.04	− 0.14	− 0.08	− 0.06	− 0.06	− 0.05	− 0.01
Severe Anaemia	− 0.02	− 0.006	− 0.014	− 0.0013	− 0.007	− 0.006	− 0.037	− 0.016	−0.021

Source: Authors’ estimates based on unit level data of NFHS-3 and NFHS-4

*estimated at 95% CI and *p*-value< 0.05

**Note: ‘(−) negative sign’ signifies reduction in SII values from 2005 to 06 to 2015–16

### Nutrition status of women (15–49 years) and children (under 5) according to wealth quintile

**Fig. 2:**
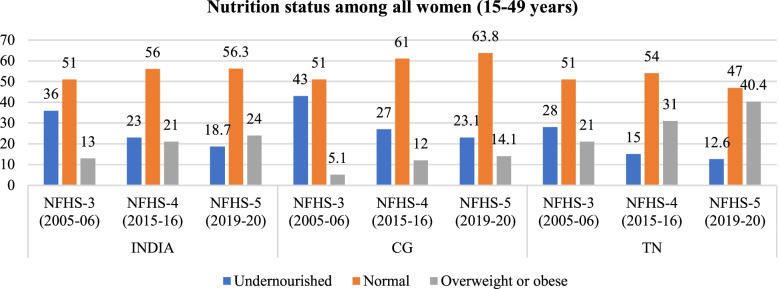
Trends of indicators of nutrition (BMI) among women in India, CG, TN from NFHS-3 (2005–06) to NFHS-5 (2019–20). Source: Data from NFHS-3, NFHS-4 and NFHS-5 fact sheet of India, CG and TN

As discussed previously the women whose BMI is lower than 18.5 are considered as acutely malnourished or ‘thin’. The proportion of ‘thin’ or acutely undernourished women has decreased across all the wealth quintiles between 2005 and 06 and 2019–20 (Fig. 2).

**Fig. 3:**
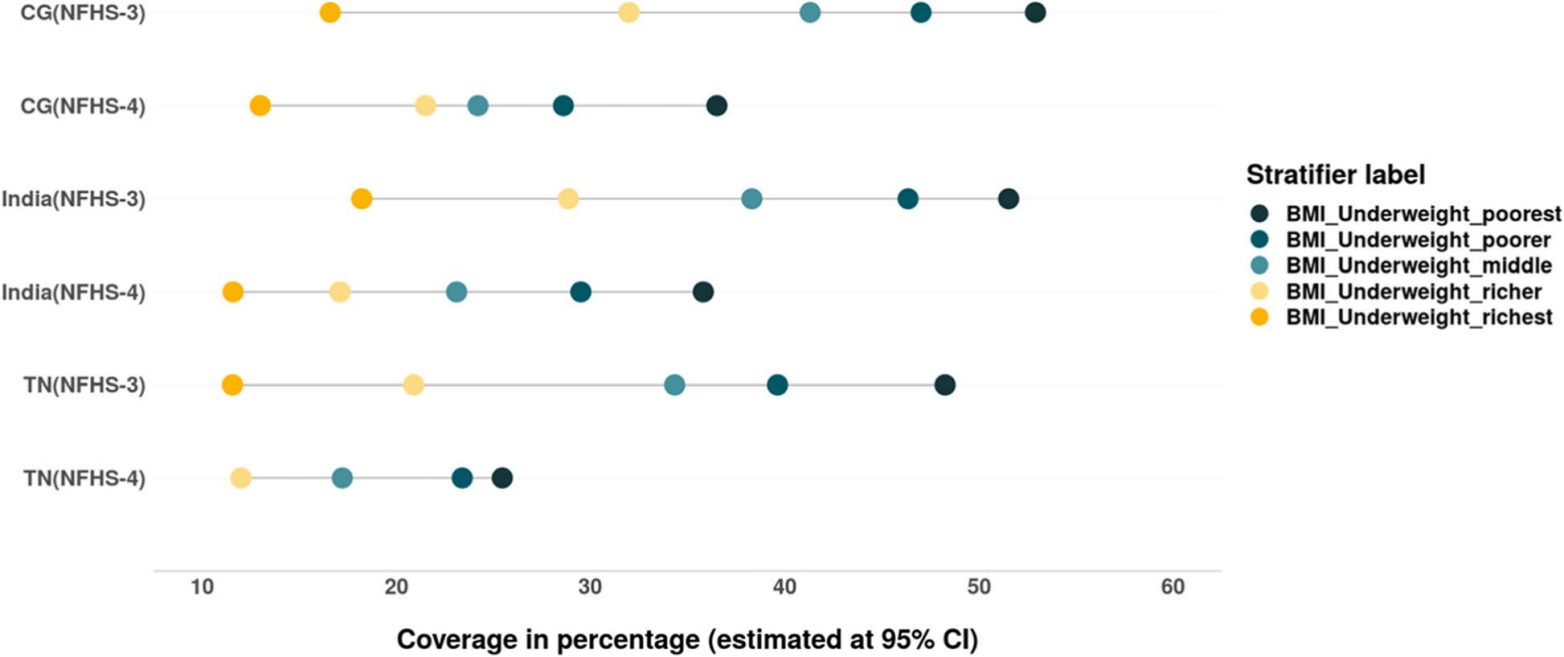
Thinness or acute undernutrition among women (BMI < 18.5). Source: Authors’ estimates based on unit level data of NFHS-3 and NFHS-4

Despite the decline in inequality between the wealth quintiles, undernutrition is still concentrated among women belonging to poor households (Fig. 3 Tables 4 & 5). The most marked reduction across all the wealth quintiles was observed in TN (Fig. 3).

**Table 5:** Trends in wealth related relative inequality (CIX) in prevalence of malnutrition and anaemia among women

CIX	India	India	Change in relative inequality **	CG	CG	Change in relative inequality**	TN	TN	Change in relative inequality**
Maternal nutrition indicators	NFHS-3 (2005–06)*	NFHS-4 (2015–16)*		NFHS-3 (2005–06)*	NFHS-4 (2015–16)*		NFHS-3 (2005–06)*	NFHS-4 (2015–16)*	
Thin	−0.21	− 0.19	−0.02	− 0.158	−0.148	− 0.010	−0.26	− 0.182	−0.078
Normal	0.008	−0.02	−0.01	0.6	−0.014	− 0.586	−0.029	− 0.037	0.008
Overweight/obesity	0.388	0.31	−0.078	0.61	0.216	−0.384	0.335	0.31	−0.025
anaemia	−0.068	−0.04	− 0.028	−0.075	− 0.073	−0.003	− 0.048	−0.028	− 0.020
Mild anaemia	−0.049	− 0.033	−0.016	− 0.053	−0.059	− 0.006	−0.024	− 0.014	−0.010
moderate anaemia	−0.113	−0.058	− 0.055	−0.143	− 0.126	−0.017	− 0.077	−0.052	− 0.025
Severe anaemia	−0.182	− 0.089	−0.093	− 0.0324	−0.117	− 0.0846	−0.296	− 0.176	−0.12

Source: Authors’ estimates based on unit level data of NFHS-3 and NFHS-4

*estimated at 95% CI and *p*-value< 0.05

**Note: ‘(−) negative sign’ signifies reduction in CIX values from 2005 to 06 to 2015–16

**Fig. 4:**
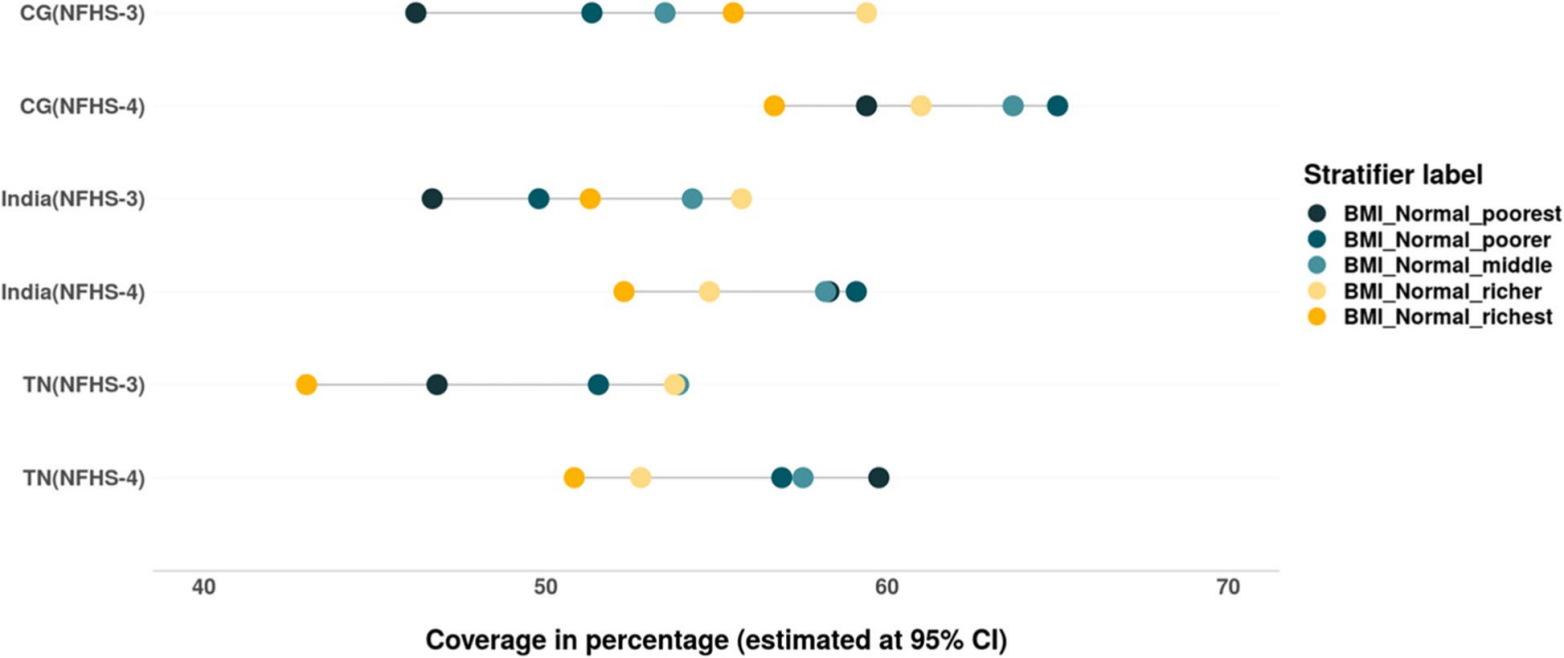
Trends in wealth related inequality among women with healthy BMI. Source: Authors’ estimates based on unit level data of NFHS-3 and NFHS-4

The wealth related gap among the women from different wealth quintiles with the normal BMI, has narrowed in the past decade at national level as well as in TN, CG. The trend has changed in the past decade, where a higher proportion of women from poor household have BMI within normal range as compared to their rich counterparts. The change was quite distinct in India and CG. In TN, across both the rounds, a higher proportion of women belonging to poor households have normal BMI as compared to their richer counterparts (Fig. 4).

**Fig. 5:**
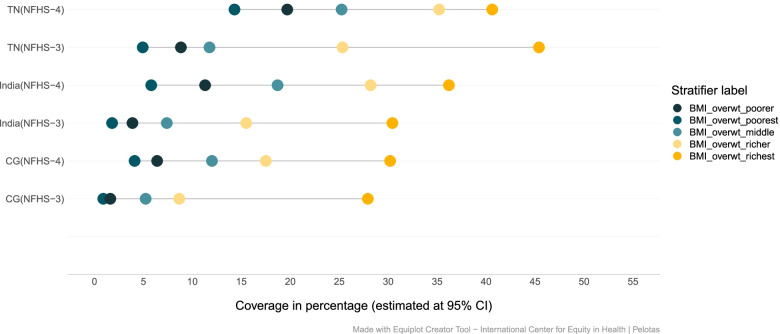
Trends in wealth related inequality in prevalence of overweight or obesity (BMI > 25.0 to 30.0; BMI > 30.0 respectively) among women of reproductive age (15–49 years). Source: Authors’ estimates based on unit level data of NFHS-3 and NFHS-4

The prevalence of overweight and obesity has increased among women from all economic backgrounds in India, CG and TN. The prevalence of overweight or obesity among women from poor households has increased from 2% (2005–06) to 6% (2015–16). While the prevalence among women from wealthier households has increased from 30.43%(2005–06) to 36.2% (2015–16). The changing trends in inequality indicate that higher proportion of women from poor households are falling into obese or overweight categories when compared to 2005–06, this change is quite prominent in TN. However, the burden of obesity/overweight remains higher among women from rich households (Fig. 5, Tables 4 & 5). Women in either of the extreme body weight categories (underweight and overweight) may undergo adverse perinatal outcomes, infertility, preterm birth and even neonatal mortality.

### Prevalence of anaemia among women of reproductive age (15–49 years) across wealth quintiles

**Fig. 6:**
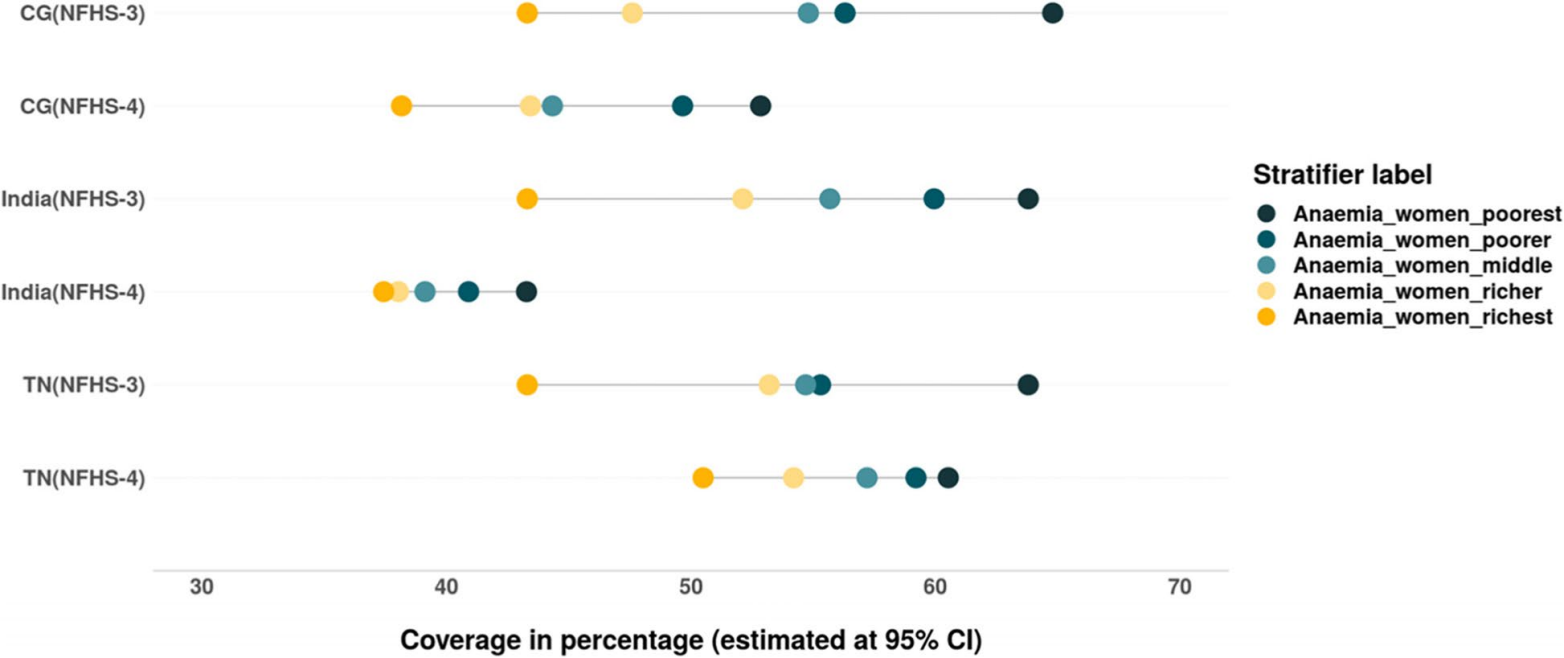
Trends in wealth related inequality in prevalence of anaemia among women of reproductive age (15–49 years) of different wealth quintiles. Source: Authors’ estimates based on unit level data of NFHS-3 and NFHS-4

The prevalence of anaemia has reduced across all the wealth quintiles at national level. Between 2005–06 and 2015–16 a greater reduction is evidenced among women from poor households. However, the prevalence of anaemia shows pro poor inequality pattern (Tables 4 & 5). The prevalence of anaemia has increased amongst the women from rich households in TN between 2005–06 and 2015–16 (Fig. 6).

**Fig. 7:**
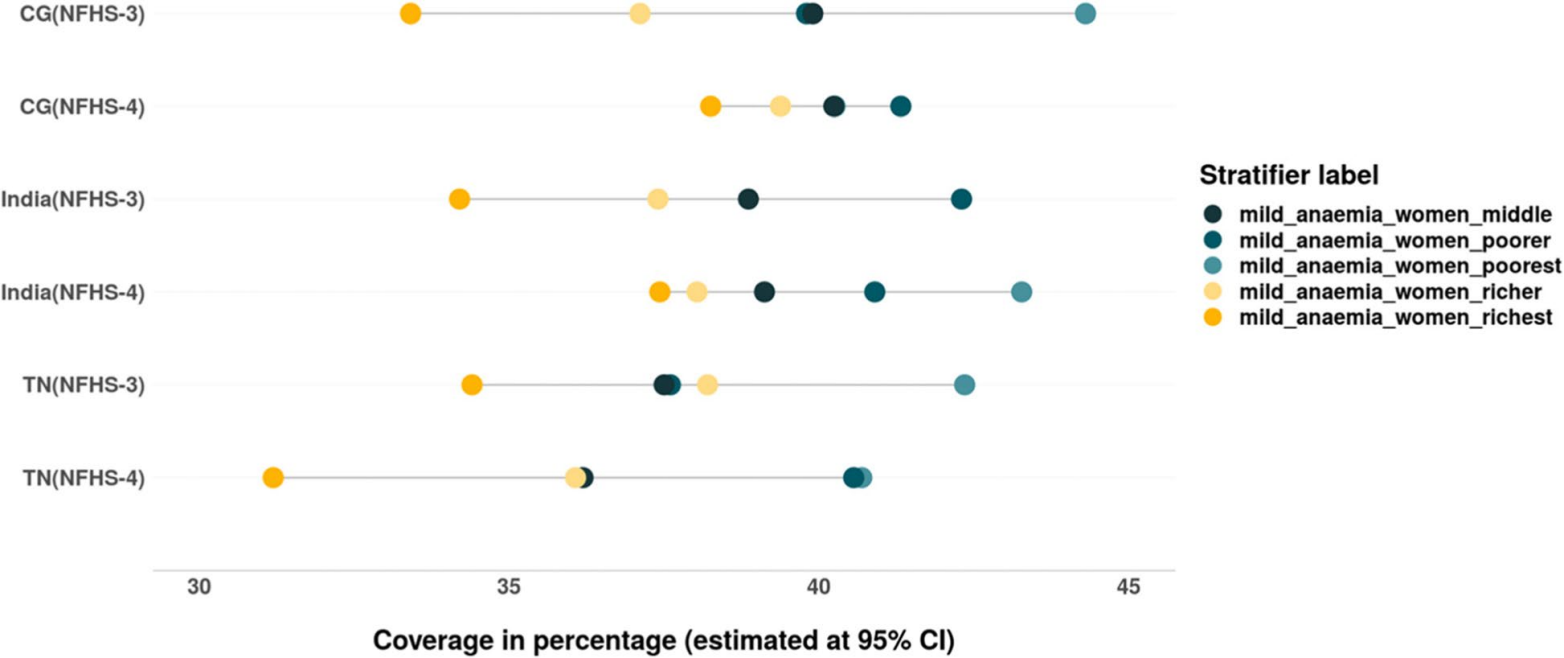
Trends in wealth related inequality in prevalence of mild anaemia (Hb-10-11.9 g/dL) among women of reproductive age (15–49 years) of different wealth quintiles. Source: Authors’ estimates based on unit level data of NFHS-3 and NFHS-4

**Fig. 8:**
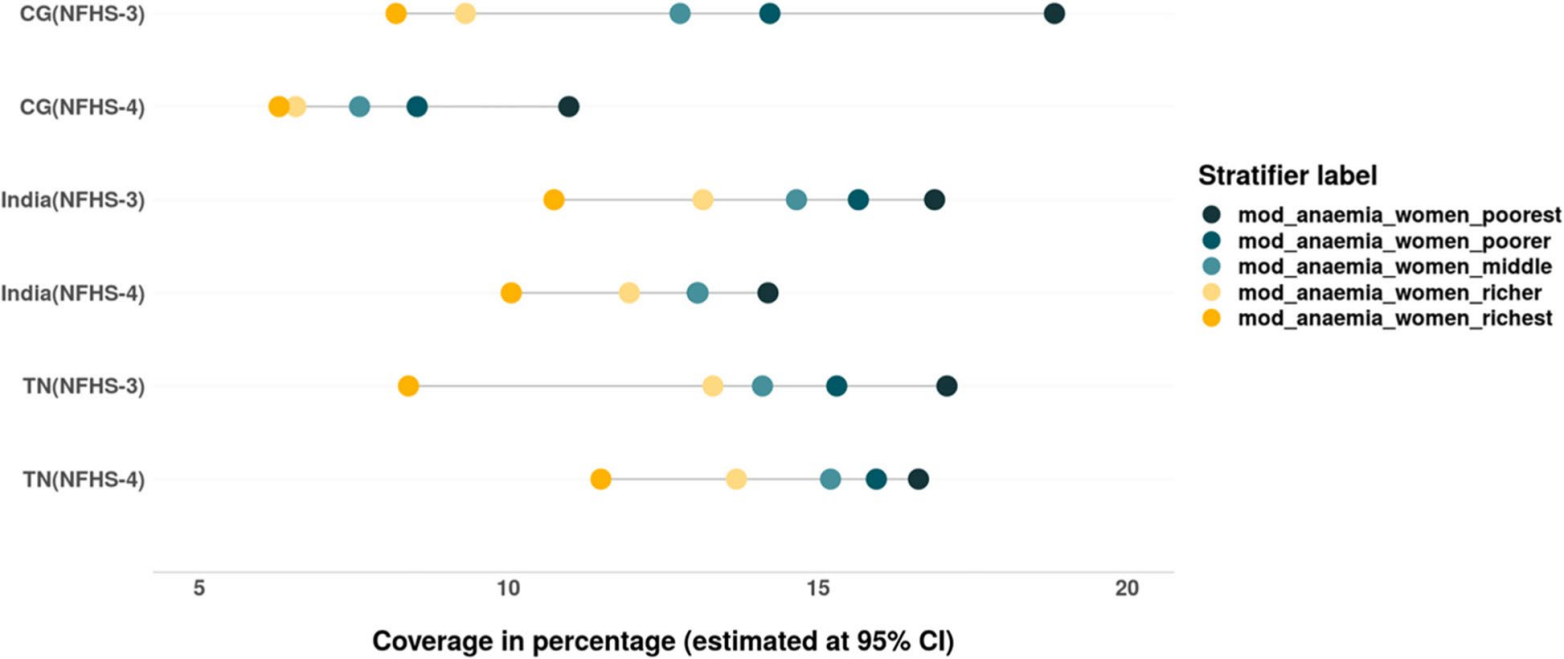
Trends in wealth related inequality in prevalence of moderate anaemia among women (Hb- 7.0 g/dL to 9.9 g/dL) among women of reproductive age (15–49 years) of different wealth quintiles. Source: Authors’ estimates based on unit level data of NFHS-3 and NFHS-4

**Fig. 9:**
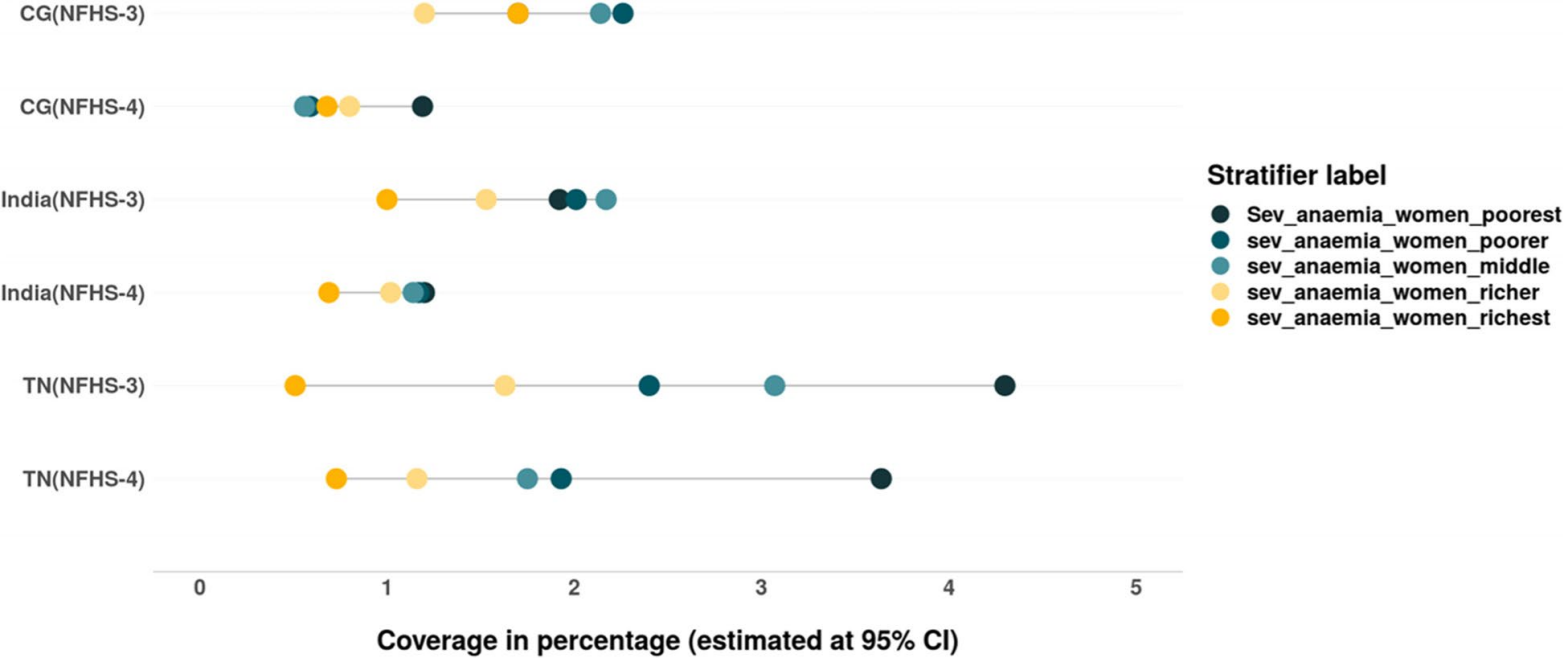
Trends in wealth related inequality in prevalence of severe anaemia among women (Hb < 7.0 g/dl) among women of reproductive age (15–49 years) of different wealth quintiles. Source: Authors’ estimates based on unit level data of NFHS-3 and NFHS-4

We also assessed the inequalities in prevalence of all three forms of anaemia – mild, moderate and severe among women belonging to different wealth quintiles (Tables 4 & 5). The magnitude as well as inequality in prevalence of different forms of anaemia across the wealth quintiles has reduced in the past decade in India, CG and especially in TN, where the prevalence of anaemia across the wealth quintiles is lower than that at the national level. However, the converging pattern evidenced in the prevalence of mild anaemia in India and CG is not only due to the decline among women from poor household but also due to the increase in prevalence of mild anaemia among women from rich households (Fig. 7). There is slight increase in prevalence of moderate form of anaemia among women from richest households between 2005–06 and 2015–16 in TN (Fig. 8). The gap between the wealth quintiles is least with regards to the prevalence of severe anaemia (Fig. 9; Tables 4 & 5).

### Trends in nutritional status and anaemia among children (under 5) according to wealth quintile

**Fig. 10:**
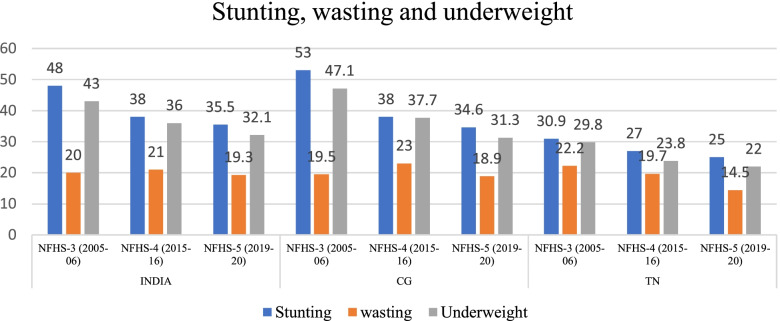
Trends in stunting, wasting and underweight among children (6–59 months) in India, CG and TN. Source: Data from NFHS-3, NFHS-4 and NFHS-5 fact sheet

**Fig. 11:**
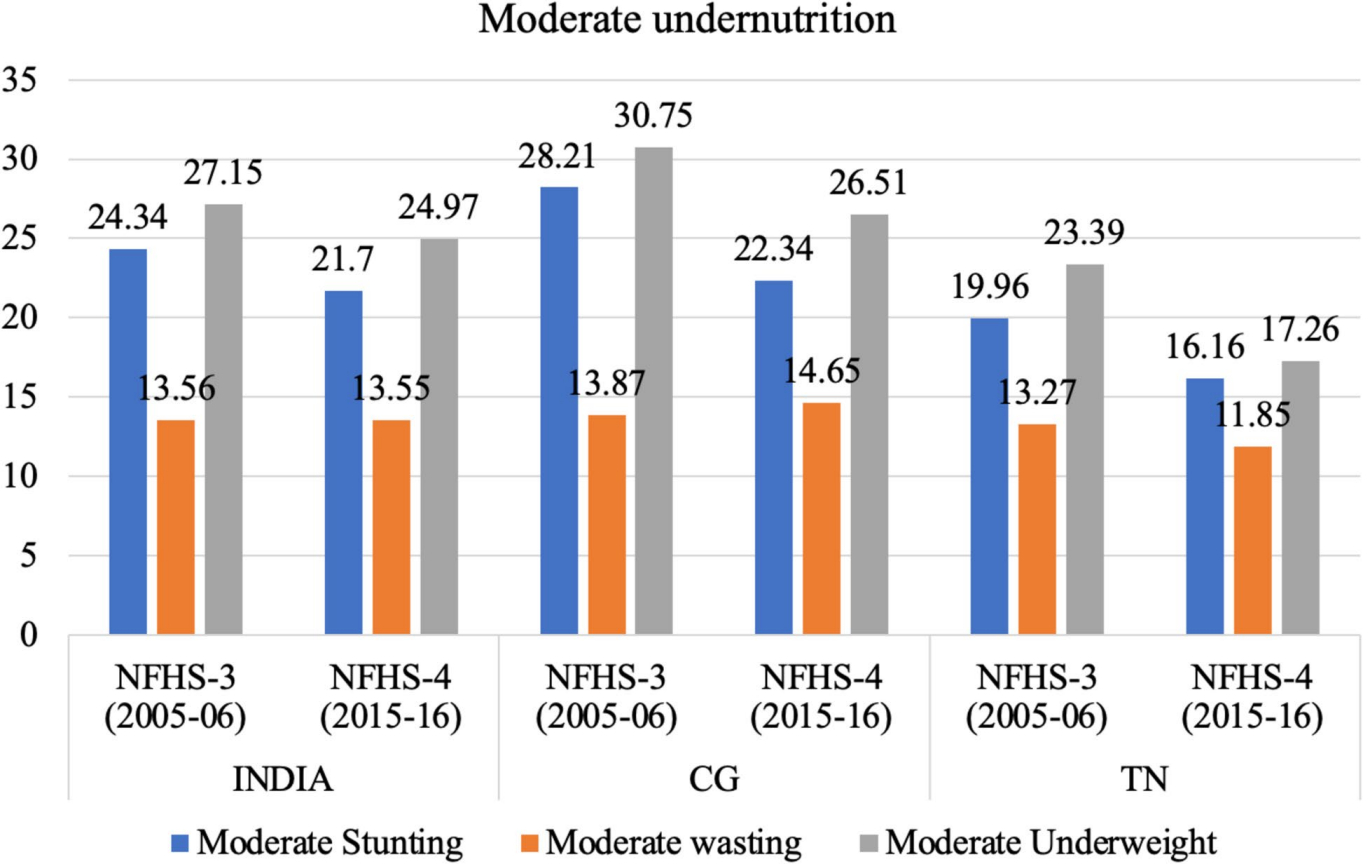
Trends in moderate form of stunting, wasting and underweight among children (6–59 months) in India, CG and TN (includes children between − 2 & -3 standard deviations (SD) from the WHO Child Growth standards population median). Source: Author’s estimates based on unit level data of NFHS-3 and NFHS-4 and data from NFHS-5 fact sheet

The prevalence of stunting and underweight among under 5 children has reduced between 2005–06 and 2015–16 in the two states and at the all India level but the prevalence of wasting has increased in India and CG during this period (Fig. 10). Moderate malnutrition also shows the same trend as above, except for reduction in prevalence of wasting in TN (Fig. 11). The prevalence of stunting, wasting as well as underweight has declined between 2015–16 and 2019–20 at national level as well as in CG and TN.

**Fig. 12:**
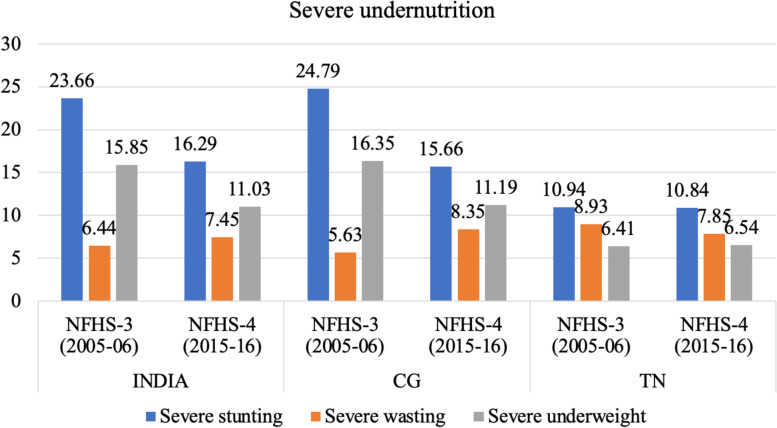
Trends in prevalence of severe form of stunting, wasting and underweight among children from NFHS-3 (2005–06) to NFHS-4 (2015–16) (only those children who are below-3 standard deviations (SD) from the WHO Child Growth Standards population median). Source: Data from NFHS-3, NFHS-4 and NFHS-5 fact sheet

The prevalence of severe form of stunting and underweight has reduced between 2005–06 and 2015–16 in India and CG while severe wasting has shown an upward trend. In TN, prevalence of severe wasting has reduced in the past decade, but prevalence of severe forms of stunting and underweight remained unchanged (Fig. 12). It is significant to note that while the levels of severe underweight and stunting in TN are much lower than the rest of the country, the level of severe wasting has been higher than the CG and all India levels in 2005–06 and continues to be higher than the latter in 2015–16 also. This is highlighted later in the Discussion section of this paper.

**Fig. 13:**
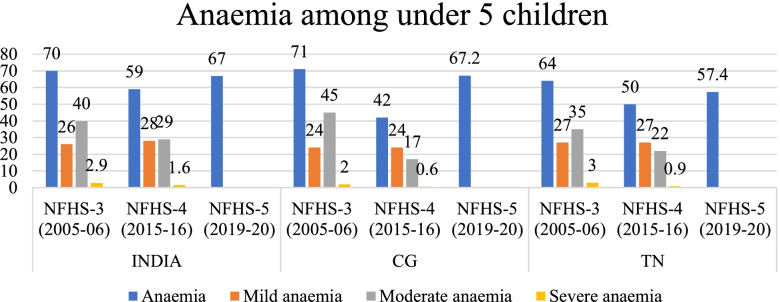
Trends in prevalence of anaemia among children in India, CG, TN from NFHS-3 (2005–06) to NFHS-4 (2015–16). Source: Data from NFHS-3, NFHS-4 and NFHS-5 fact sheet of India, CG and TN. Note: NFHS-5unit level data yet to be released, hence estimation of prevalence of different forms of anaemia for NFHS-5 round was not possible

The prevalence of anaemia among children (under 5) has reduced by 11 pp. in India while in CG and TN it has reduced by 29 pp. and 14 pp. respectively between 2005–06 and 2015–16. Between 2015–16 and 2019–20 prevalence of anaemia has increased by 8 pp. in India, 25 pp. and 7.4 pp. in CG and TN respectively (Fig. 13). The prevalence of mild anaemia among children has increased at national level, whereas it remained unchanged in CG and TN between 2005 and 06 and 2015–16. However, the prevalence of moderate and severe anaemia has reduced in the same period.

### Trends in undernutrition status of children (6–59 months) according to wealth quintile using NFHS-3 (2005–06) & NFHS-4(2015–16)

**Fig. 14:**
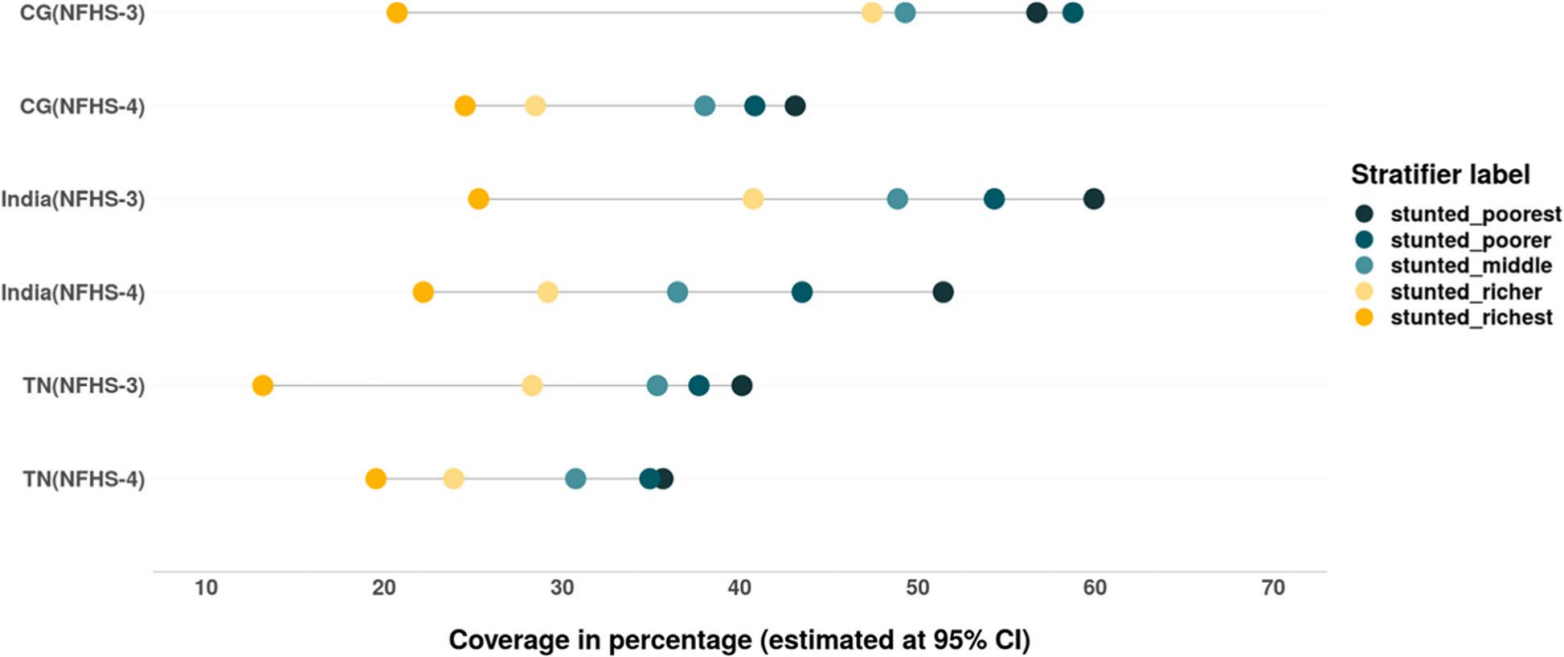
Trends in prevalence of stunting among children across wealth quintiles. Source: Authors’ estimates based on unit level data of NFHS-3 and NFHS-4

**Fig. 15:**
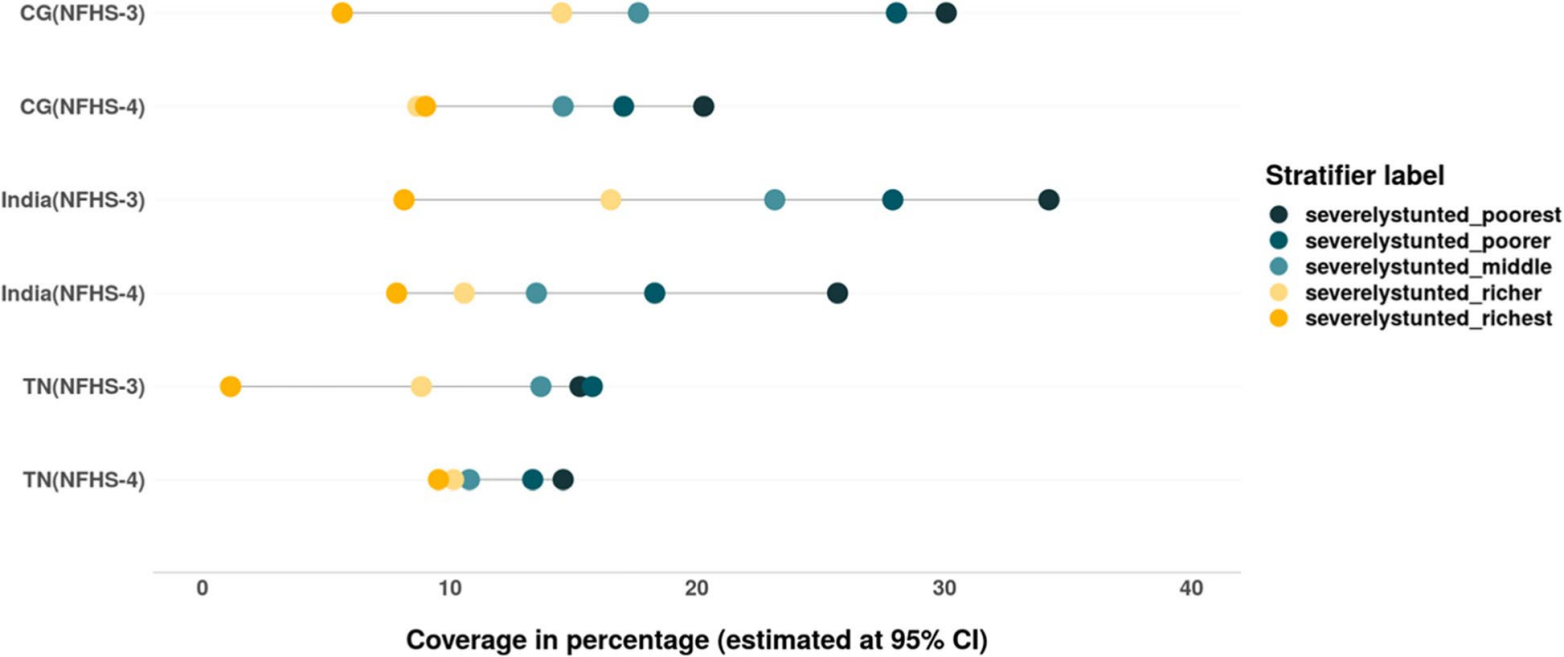
Trends in prevalence of severe stunting among children across wealth quintiles. Source: Authors’ estimates based on unit level data of NFHS-3 and NFHS-4

The overall prevalence of stunting and severe stunting has reduced between 2005 and 06 and 2015–16. The SII and CIX values suggest that the inequality among children from different wealth quintiles has also decreased in India, CG and TN (Tables 6 & 7). As evident from Fig. 14, the decline in stunting and severe stunting among children of poor households is higher than their richer counterparts in India, CG and TN. However, there is increase in prevalence of stunting as well as severe stunting among children from wealthiest quintile in CG as well as TN during this period (Figs. 14 & 15).

**Table 6:** Change in wealth related absolute inequality (SII) in undernutrition and anaemia among children (under 5) between 2005 and 06 and 2015–16

SII	India	India	Change in absolute inequality **	CG	CG	Change in absolute inequality **	TN	TN	Change in absolute inequality **
Child nutrition indicators	NFHS-3 (2005–06)*	NFHS-4 (2015–16)*		NFHS-3 (2005–06)*	NFHS-4 (2015–16)*		NFHS-3 (2005–06)*	NFHS-4 (2015–16)*	
Stunting	−0.40	− 0.338	− 0.062	− 0.288	− 0.235	− 0.053	− 0.304	− 0.192	− 0.112
Severe stunting	− 0.31	− 0.223	− 0.087	− 0.276	− 0.172	− 0.104	−0.159	− 0.060	− 0.099
Wasting	− 0.143	−0.099	− 0.044	− 0.128	− 0.139	0.011	− 0.158	− 0.035	− 0.193
severe wasting	− 0.052	− 0.040	− 0.012	− 0.172	− 0.072	− 0.100	− 0.097	− 0.005	− 0.092
Underweight	−0.423	− 0.339	− 0.084	−0.393	− 0.289	−0.104	− 0.318	−0.200	− 0.118
severe underweight	−.232	−0.164	− 0.068	−.22	− 0.15	− 0.07	−0.09	− 0.05	−0.04
Anaemia	−0.26	−0.12	− 0.14	−0.21	− 0.14	−0.07	− 0.17	−0.11	− 0.06
Mild anaemia	−0.04	− 0.033	−0.367	0.003	−0.06	0.063	−0.016	− 0.03	0.024
Moderate anaemia	−0.21	−0.096	− 0.114	−0.22	− 0.07	−0.15	− 0.12	−0.08	− 0.04
Severe anaemia	−0.02	0.0002	−0.200	0.002	0.001	−0.001	−0.04	− 0.003	−0.037

Source: Authors’ estimates based on unit level data of NFHS-3 and NFHS-4

*estimated at 95% CI and *p*-value< 0.05

**Note: ‘(−) negative sign**’** signifies reduction in SII values from 2005 to 06 to 2015–16

**Table 7:** Change in wealth related relative inequality (CIX) in undernutrition and anaemia among children (under 5) between 2005 and 06 and 2015–16

Child nutrition indicators	India	India	Change in relative inequality **	CG	CG	Change in relative inequality **	TN	TN	Change in relative inequality **
	NFHS-3 (2005–06)*	NFHS-4 (2015–16)*		NFHS-3 (2005–06)*	NFHS-4 (2015–16)*		NFHS-3 (2005–06)*	NFHS-4 (2015–16)*	
Stunting	−0.15	− 0.14	− 0.01	−0.083	− 0.093	0.010	− 0.156	−0.109	− 0.047
Severe stunting	−0.235	− 0.21	−0.025	− 0.174	−0.152	− 0.022	−0.233	− 0.090	−0.143
Wasting	−0.133	−0.08	− 0.053	−0.099	− 0.08	−0.018	− 0.117	−0.023	− 0.094
severe wasting	−0.140	− 0.09	−0.05	− 0.092	−0.105	0.013	−0.166	− 0.011	−0.155
Underweight	−0.192	−0.157	− 0.035	−0.131	− 0.120	−0.011	− 0.172	−0.109	− 0.063
severe underweight	−0.282	− 0.235	−0.047	− 0.204	−0.173	− 0.031	−0.217	− 0.110	−0.107
Anaemia	−0.071	−0.033	− 0.038	−0.049	− 0.043	−0.006	− 0.043	−0.039	− 0.004
Mild anaemia	−0.024	− 0.022	−0.002	− 0.004	−0.039	− 0.001	−0.027	− 0.024	−0.003
Moderate anaemia	−0.103	−0.047	− 0.056	−0.075	− 0.051	−0.024	− 0.043	−0.054	0.011
Severe anaemia	−0.101	0.018	−0.083	−0.006	− 0.002	−0.004	− 0.23	−0.085	− 0.145

Source: Authors’ estimates based on unit level data of NFHS-3 and NFHS-4

*estimated at 95% CI and *p*-value< 0.05

**Note: ‘(−) negative sign’ signifies reduction in CIX values from 2005 to 06 to 2015–16

**Fig. 16:**
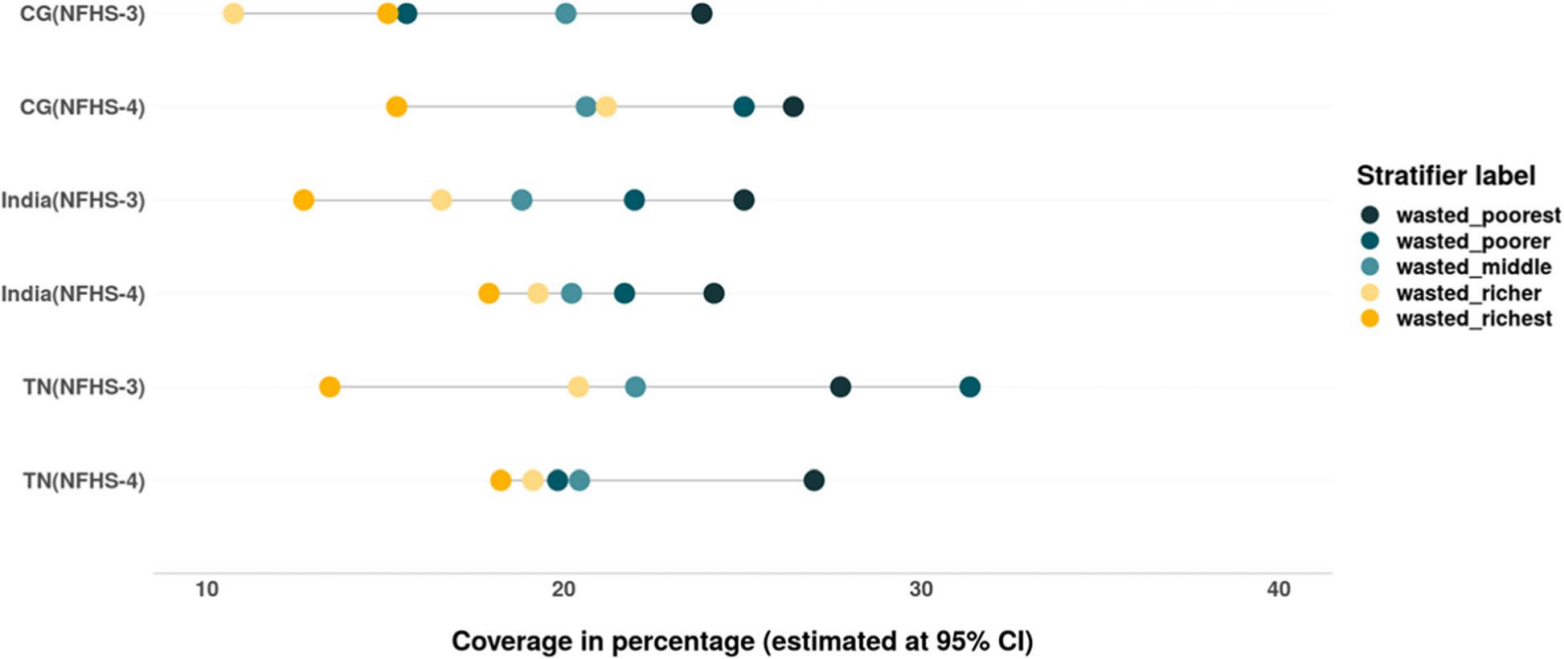
Trends in prevalence of wasting among children across wealth quintiles. Source: Authors’ estimates based on unit level data of NFHS-3 and NFHS-4

**Fig. 17:**
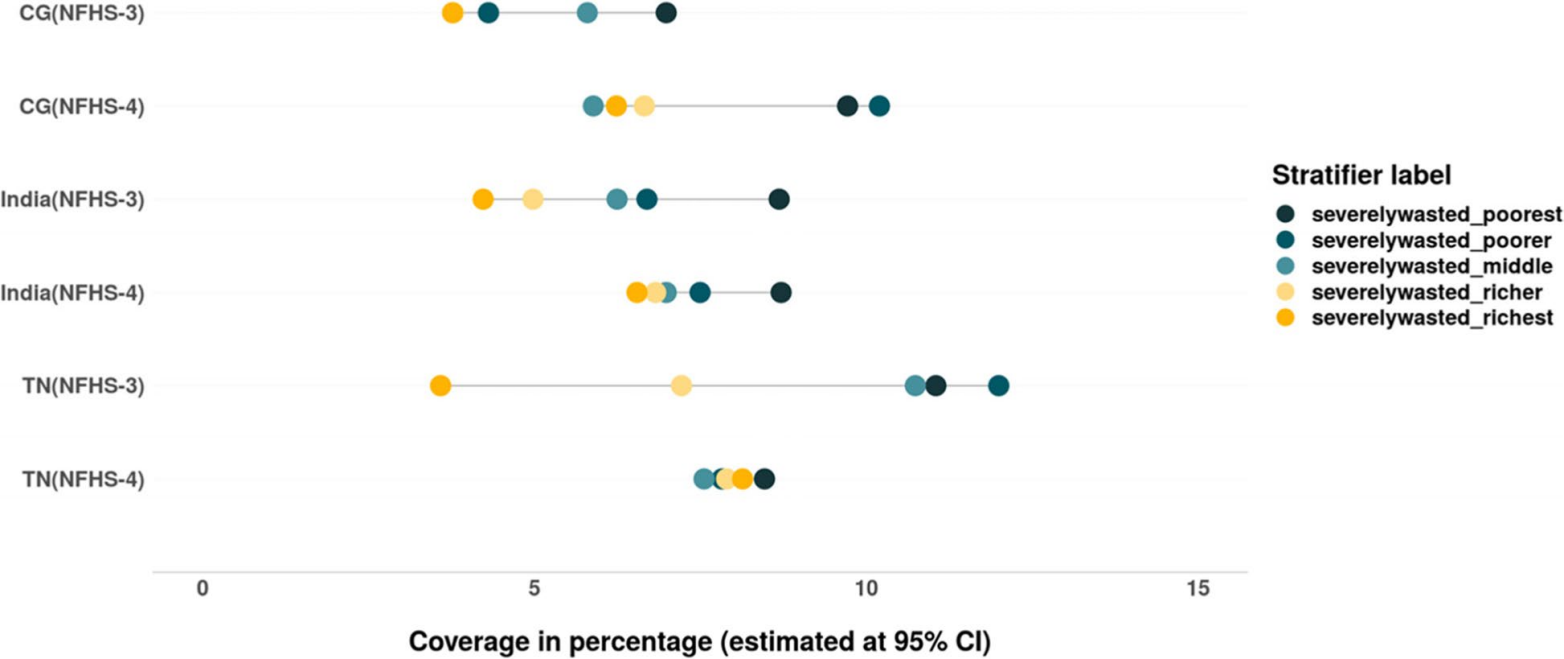
Trends in prevalence of severe wasting among children across wealth quintiles. Source: Authors’ estimates based on unit level data of NFHS-3 and NFHS-4

The prevalence of wasting as well as severe wasting has increased in India and CG between 2005 and 06 and 2015–16 but not in TN. The CIX and SII values for wasting and severe wasting suggest that inequality has reduced in CG, TN and India. Maximum reduction in the relative and absolute inequality values (9 pp. and above) was evidenced in TN between 2005 and 06 and 2015–16 (Tables 6 & 7). The prevalence of wasting as well as severe wasting as observed in the case of stunting has increased among the children from rich households in TN as well as CG (Figs. 16 & 17).

**Fig. 18:**
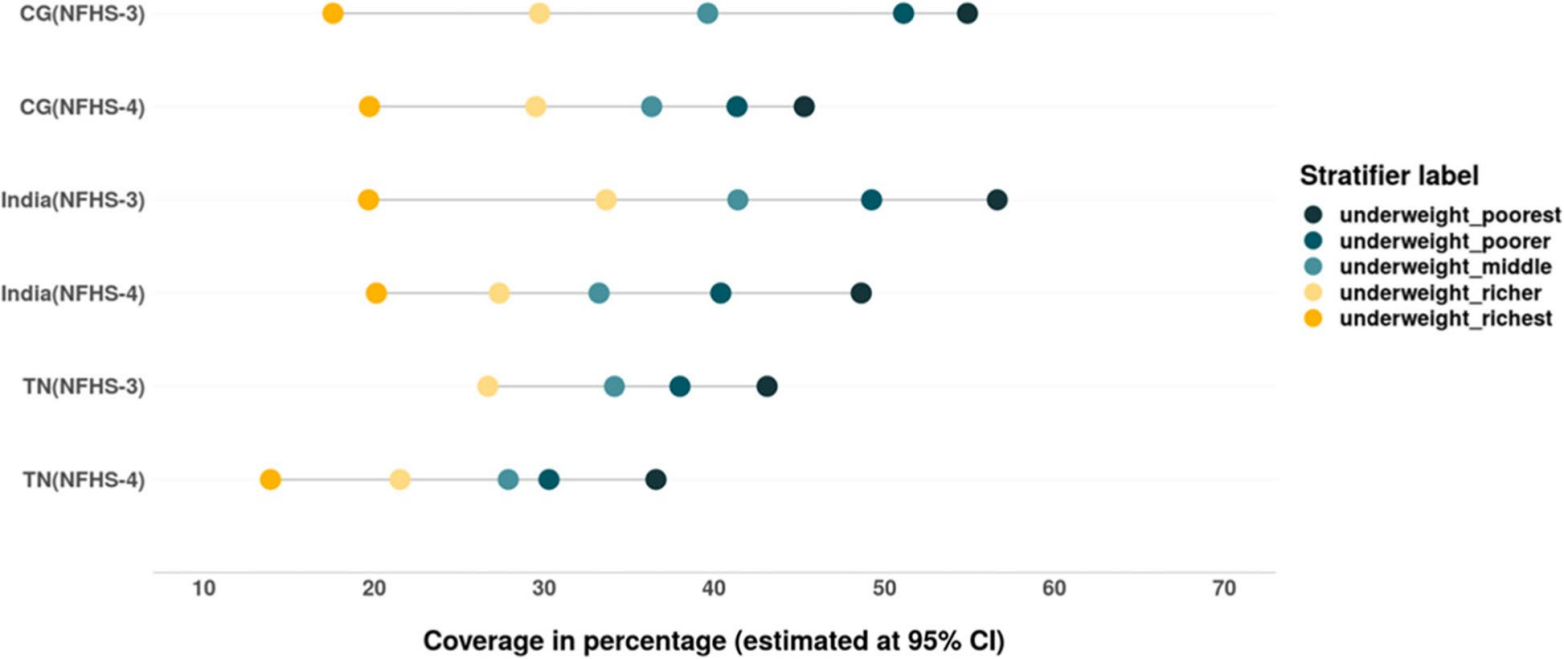
Trends in prevalence of underweight among children across wealth quintiles between. Source: Authors’ estimates based on unit level data of NFHS-3 and NFHS-4

**Fig. 19:**
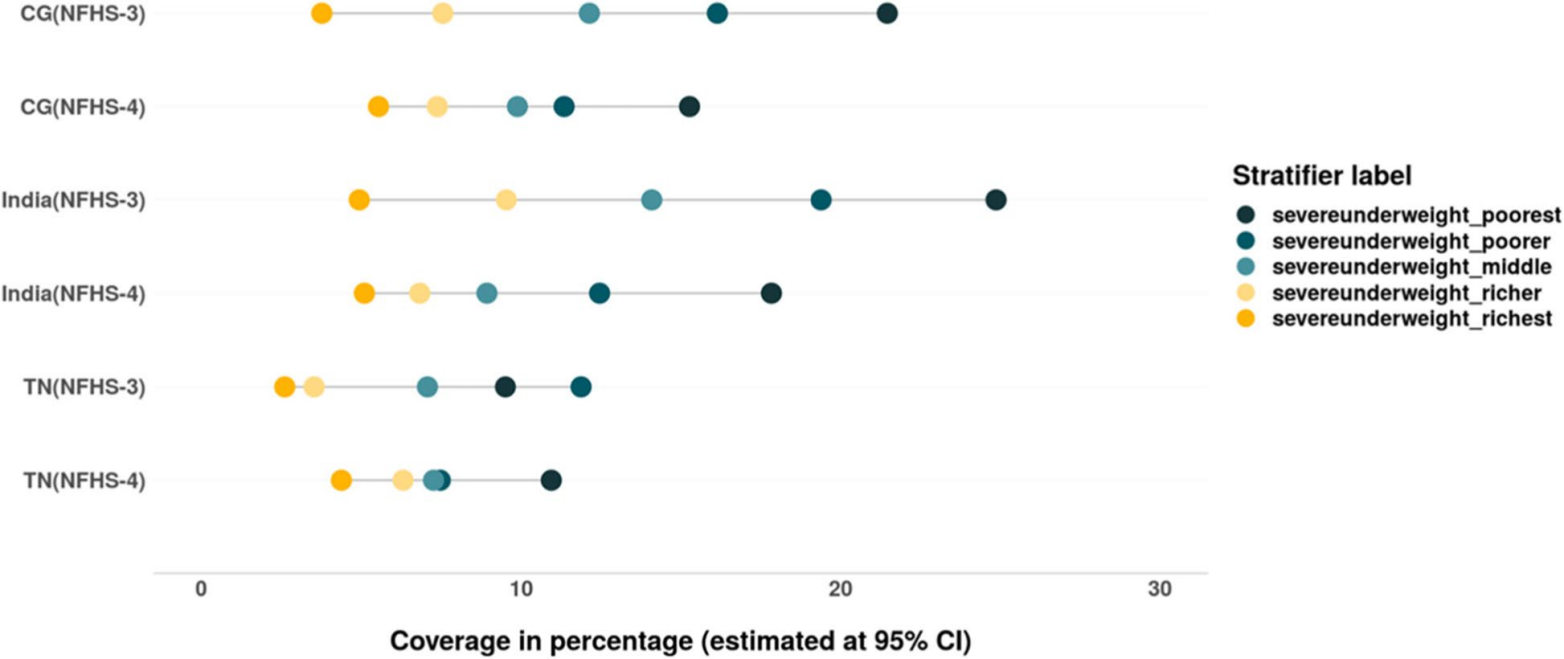
Trends in prevalence of severe underweight among children across wealth quintiles. Source: Authors’ estimates based on unit level data of NFHS-3 and NFHS-4

Similar to the pattern of prevalence of stunting and wasting among children (under 5), the wealth gaps with regards to prevalence of underweight has also reduced. The prevalence of underweight (in CG) and it’s severe form (in both CG and TN) among children from rich households has increased between 2005–06 and 2015–16, however a decline was observed among their poor counterparts (Figs. 18 & 19). Though the absolute and relative inequalities have reduced, but the burden of underweight and severe underweight is still concentrated among children belonging to poor households (Tables 6 & 7).

**Fig. 20:**
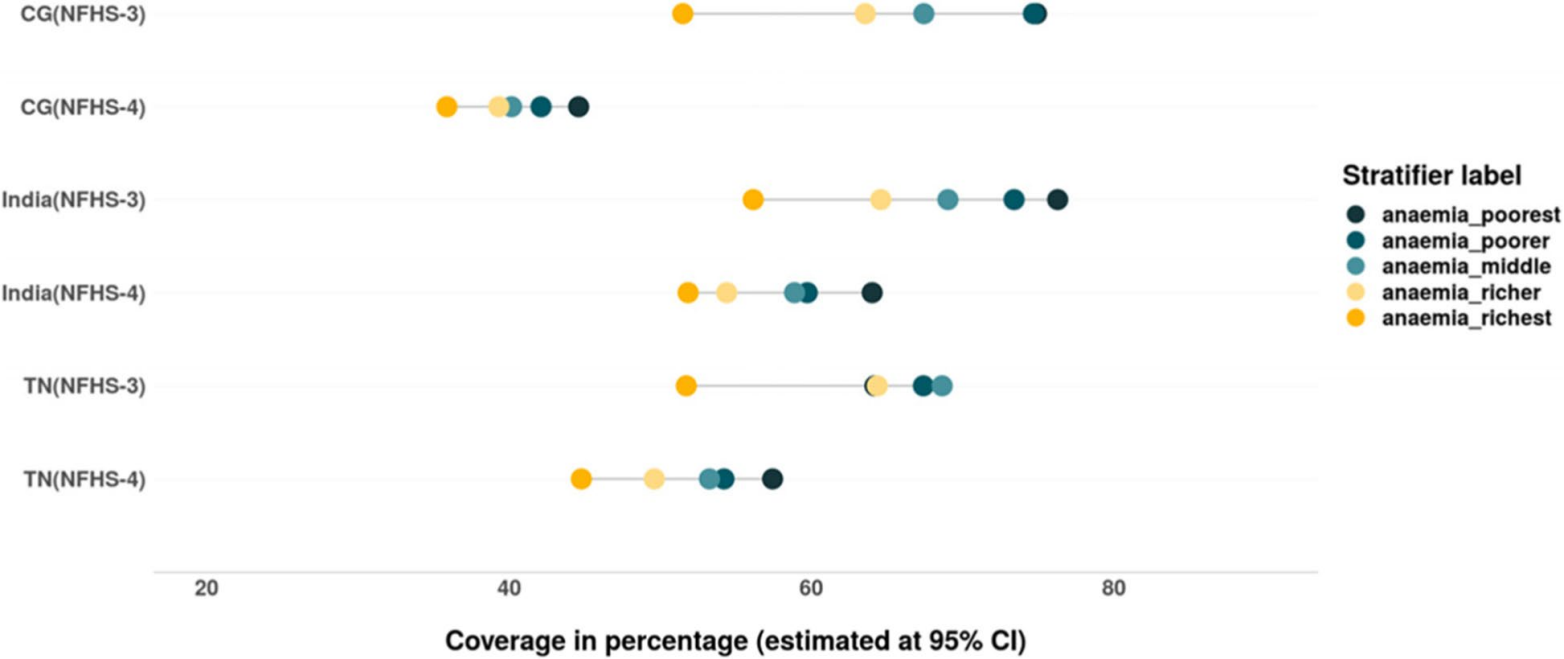
Trends in prevalence of anaemia among children across wealth quintiles. Source: Authors’ estimates based on unit level data of NFHS-3 and NFHS-4

**Fig. 21:**
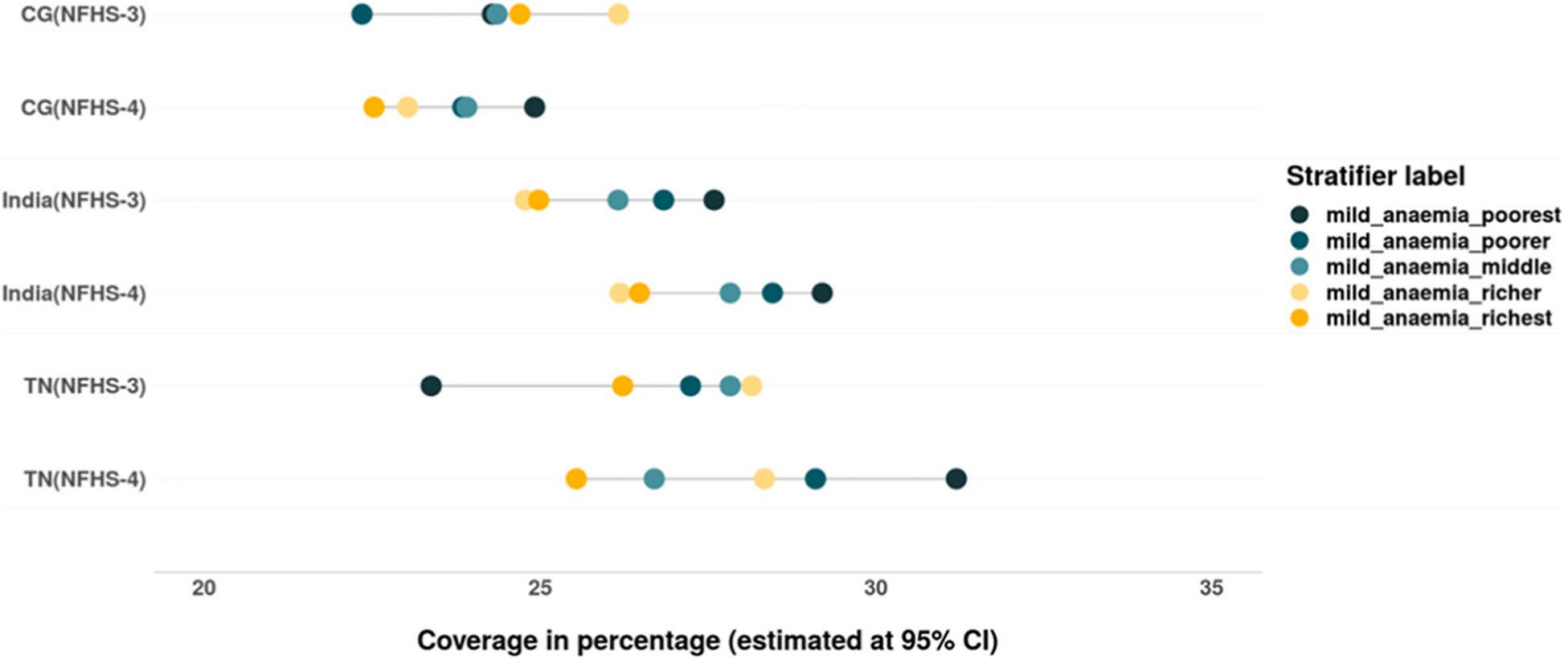
Trends in prevalence of mild anaemia among children across wealth quintiles. Source: Authors’ estimates based on unit level data of NFHS-3 and NFHS-4

**Fig. 22:**
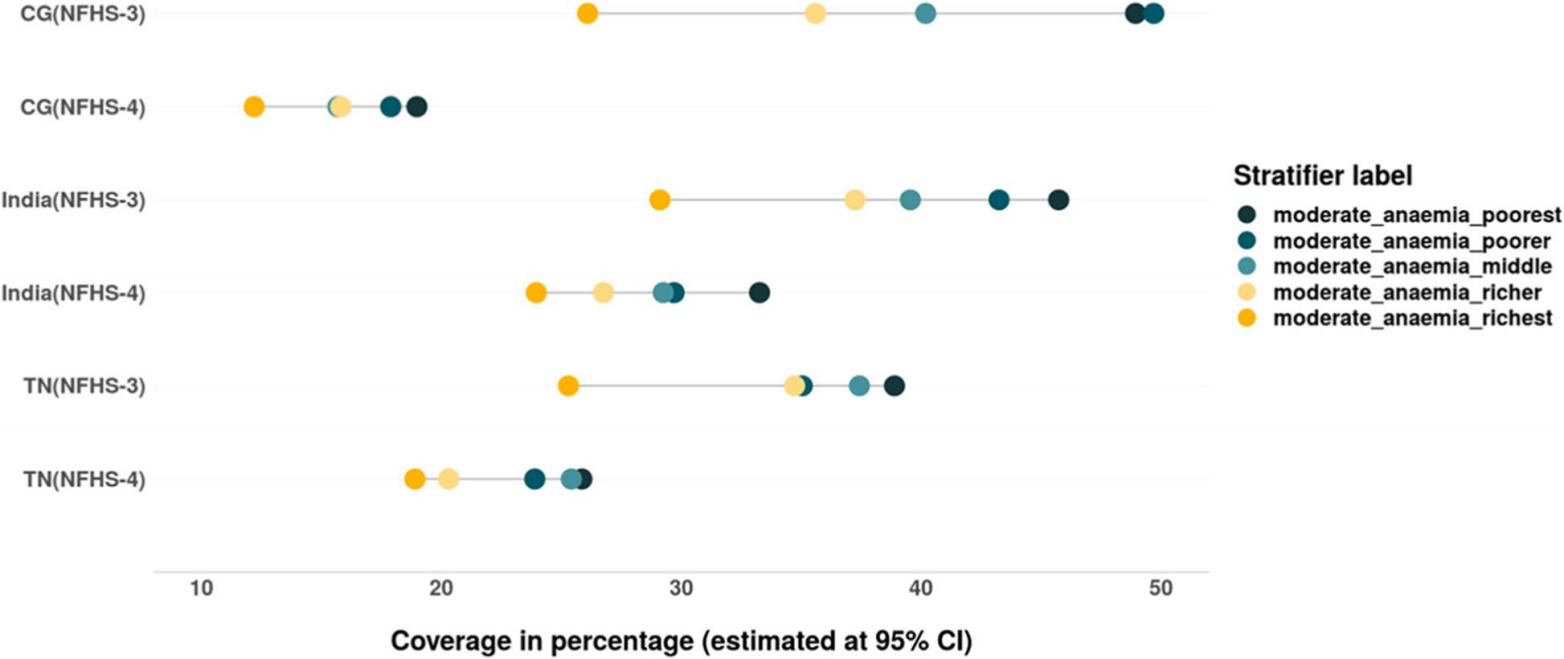
Trends in prevalence of moderate anaemia among children across wealth quintiles. Source: Authors’ estimates based on unit level data of NFHS-3 and NFHS-4

**Fig. 23:**
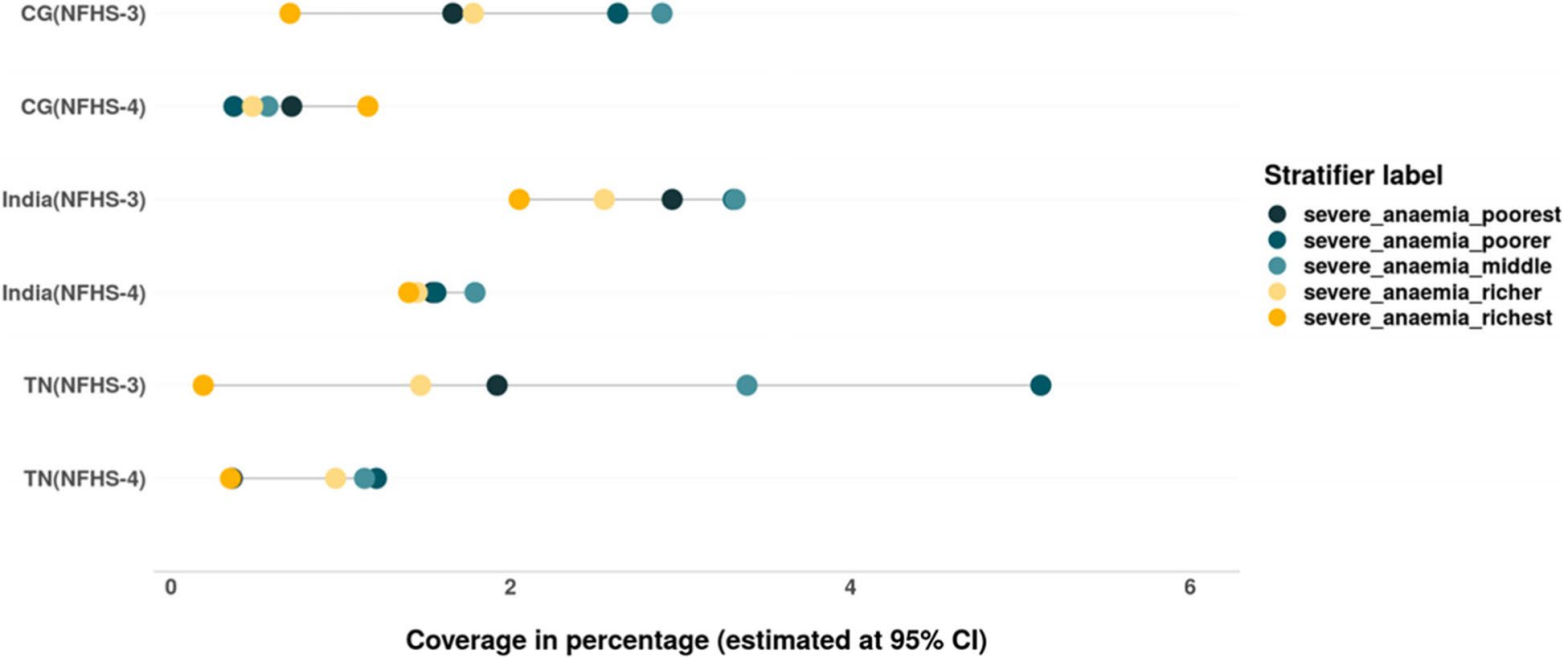
Trends in prevalence of severe anaemia among children of different wealth quintiles. Source: Authors’ estimates based on unit level data of NFHS-3 and NFHS-4

The prevalence of anaemia among children has reduced between 2005–06 and 2015–16 in India, CG and TN (Fig. 20). There is a reduction in inequality in prevalence of anaemia (mild, moderate and severe), the burden of anaemia is still higher among the children belonging to poor households (Tables 6 & 7). There is an increase in prevalence of mild forms of anaemia among children from rich households at all India level. While an increase in prevalence of moderate anaemia was observed among the children from poor households in TN, CG and India and among children from rich households at all India level. Remarkable reduction in prevalence as well as wealth gap was observed with regards to moderate anaemia among children in India, TN as well as CG (Fig. 21 & 22). Inequality was least with respect to severe form of anaemia i.e., children from all wealth quintiles are affected by severe anaemia (Fig. 23).

## Discussion

The findings of our study indicate that though the status of maternal and child malnutrition has improved during 2005–06 and 2015–16, the reduction was uneven across wealth quintiles [26, 43, 44]. The inequalities have reduced and there is a convergence in the prevalence of undernutrition and anaemia among children and women of all wealth quintiles. However, while there was a reduction in the prevalence of undernutrition among poor households (which is desirable) there was an increase in the prevalence of certain indicators (namely obesity and overweight) among richest quintiles (which is undesirable). Previous studies have reported that the reduction in prevalence of undernutrition between 1992 and 93 and 2005–06 was higher among the children from rich households [45]. Our findings corroborate with the findings from the previous studies, except for the reduction in underweight which was higher among children from poor households than those of wealthier households. In fact, we found an increase in prevalence of stunting and underweight among children from richest quintiles. The state of world’s children report also highlighted the high prevalence of stunting among the children of wealthy households [46]. A high proportion of children from richest households were found to be stunted in Zimbabwe [47]. There is not enough literature which explains the prevalence of undernutrition in children from wealthy households. The lack of food diversity and inadequate consumption of essential nutrients even in children from rich households could be a plausible reason for prevalence of stunting even in the wealthiest quintile [48]. Children under the age of two, whether breastfed or not, do not receive the necessary nutrition, regardless of household income. According to the Comprehensive National Nutrition Survey (CNNS), only 6.4% of children in this age group consumed enough essential nutrients [49]. The consumption of proteins too remained low among children aged 2–4 years irrespective of the wealth quintile they belonged. Also, Infant and young child feeding (IYCF) practices such as exclusive breast feeding (EBF) are important for child’s development during the first 1000 days by reducing the risk of diarrhoeal infections among infants [50]. However, women from wealthy households have been found to less likely to practise EBF in India as well as globally [51, 52]. Poor IYCF practices could explain the prevalence of undernutrition among children of wealthy households. The burden of child undernutrition (stunting, wasting and underweight) remains concentrated among the poor, similar to the trends observed in other LMICs [41–43]. Children from low-income families are more likely to be exposed to pathogenic agents than those from higher-income families; once exposed, they are more likely to become ill due to lower resistance and lower coverage with preventive interventions. They are less likely to have access to health services once they become ill, and the quality of these services is likely to be lower, with less access to life-saving treatments. As a result, children from low-income families more likely to be malnourished [53, 54]. Our study also shows that there is an increase in prevalence of severe wasting in India, CG and TN and also there has been no decline in severe stunting and underweight even in TN in the same period between 2005 and 06 and 2015–16. The reasons for increase in prevalence of wasting are not clear but the current practise of using weight among children rather than using Mid upper arc circumference (MUAC) in majority of the states as a parameter to identify undernourished children could result in missing the children who are wasted. Even if the severely acute malnourished children are identified, the course of treatment is largely curative, and not preventive which could leave the underlying reasons unaddressed which caused wasting at the first place [55].

Prevalence of malnutrition among women in India follows trends similar to other low- to middle-income countries (LMIC) where the prevalence of underweight has declined considerably among women over the span of 10 years [56, 57], Though the burden of acute undernutrition is concentrated among the women belonging to poor households. But a positive change is that the inequality between the rich and poor quintile has reduced with respect to acute undernutrition between 2005 and 06 and 2015–16. Also, a higher proportion of women from poor households have normal BMI than the well-off women according to NFHS-4 at national level. A similar pattern is evidenced in TN and CG too, in TN even during 2005–06 a higher proportion of women from poor households had healthy BMI than their rich counterparts. However, the burden of overweight is still concentrated among women from the rich households. This is similar to the trends of obesity and overweight observed at global, national and regional level, where women from wealthier households were at increased risk of BMI > 25.0 kg/m^2^ and lower risk of undernutrition [6, 22, 58, 59]. India like other LMIC such as Bangladesh and Nepal has been facing the dual problem of underweight and overweight [6, 60, 61]. India is currently in the first and second stages of a nutrition transition [62, 63]. According to this theory overweight emerges first among the wealthy and urban before spreading to the rural and poor [16]. On the other hand, in ‘underweight states’, overweight and obesity have remained socially segregated and increasing among urban and richer section of the population [16]. Our study findings were also similar to the aforementioned study findings, where an increase in overweight or obesity was observed among women from poor households in TN while it increased among the women from rich households in CG. Lower nutritional outcomes in India have been linked to a preference for locally abundant foods and changes in their relative prices during economic development [64, 65]. As structural transformation progresses, more people participate in less strenuous labour activities, and total calorie requirements decrease [66]. If people’s eating habits or proclivity to exercise do not change, this could lead to even more obesity [65].

We have also assessed the wealth related inequality with respect to overall and severe forms of anaemia. Similar to other study findings, we have found rich households tend to have better nutritional status, which may be attributed to lower anaemia levels in children and women from rich households compared to their poor counterparts [26, 67–69]. There is considerable reduction in moderate forms of anaemia among children in India, CG and TN. However, the prevalence of mild forms of anaemia remains unchanged and has increased at national level. Mild and moderate forms of anaemia are concentrated more among poor children, while severe form is prevalent across children from all the wealth quintiles. With respect to prevalence of anaemia among women, inequality has reduced between the wealth quintiles but the burden of anaemia is still concentrated among the poor. An increase in prevalence of mild forms of anaemia was observed among women from rich households in India as well as CG, while in TN an increase in prevalence of moderate anaemia was observed among the women from the better off households. Least inequality existed with respect to severe forms of anaemia among women. Despite the decline in the previous decade, the prevalence of anaemia among women and children remains staggering. The changing dietary pattern such as loss of coarse cereals in the Indian diet could have contributed to this reduction in iron intake without compensation from other food groups [70].

## Conclusion

Malnutrition affects women and children across the country in various ways, ranging from undernutrition (child stunting, wasting, anaemia) to overweight and obesity. Various programmes and schemes have been launched and expanded to improve the nutritional situation of the country in the past decades- such as Integrated Child Development Scheme, Mid-day meals scheme, POSHAN (Prime Minister’s Overarching Scheme for Holistic Nutrition) Abhiyaan. They are aimed at reducing reduction of under-nutrition among women and children, particularly among the poorer section of the society and thereby reduce the overall inequality. While these programmes have had a positive impact in reducing overall malnutrition in this population and in reduction of inequality, we find that there are areas of concern. There is an increasing prevalence of wasting among children which requires a change in current policy approach. The current guidelines spell that SAM children should be followed up following the discharge from the facility to the community. The prevention aspect would necessitate collaboration with a wide range of actors, stakeholders particularly those enagaged in generation of employment, women’s education and empowerment, and other nutrition-sensitive and specific interventions. As a result, a systemic shift in how we approach the problem is required. Our study shows that women and children from better off households are also affected by malnutrition and need attention. The burden of obesity is concentrated among women from rich households, but the prevalence of obesity or overweight has increased among the women from poor households too. Also, the prevalence of anaemia among children as well as women across all wealth quintiles remains alarmingly high. The wealthiest households are assumed to be able to provide appropriate and adequate nutrition for their children, as well as access to the ‘best’ health care through private health providers. It is extremely concerning that children from wealthier households suffer from undernutrition just as much as children from poor households. The country’s malnutrition profile is changing rapidly - with improvements across several indicators of undernutrition on the one hand and rapidly rising rates of overweight/obesity on the other, particularly among adults. Therefore in order to reduce not only the inequality but the overall burden of malnutrition across various socio-economic classes in India, there is an urgent need to promptly devise appropriate strategies.

## Data Availability

The datasets generated during and/or analysed during the current study are available in the DHS and IIPS website, accessed from https://dhsprogram.com/data/available-datasets.cfm (http://rchiips.org/nfhs/districtfactsheet_NFHS-5.shtml)^33^respectively.

## Footnotes

1 Under the National Health Mission, a flagship programme of the Government of India, eight states with poor maternal and child health outcomes, (namely, Bihar, Uttar Pradesh, Jharkhand, Madhya Pradesh, Chhattisgarh, Odisha and Rajasthan) have been categorised as “high-focus states (HFS)” whereas other states with relatively better indicators have been designated as the non-high focus states [34]. A majority of states in the former group are located in the Northern parts of India, while all four states in the southern part of India are non-high focus states.
